# The Relationship Between Social Adaptation and Parenting Styles in Left-Behind and Non-Left-Behind Children: A Network Analysis

**DOI:** 10.3390/bs16060857

**Published:** 2026-05-27

**Authors:** Shuying Fu, Peng Li, Gonglu Cheng

**Affiliations:** 1 Faculty of Psychology, Tianjin Normal University, Tianjin 300387, China; fusy@yctu.edu.cn (S.F.);; 2 Jiangsu Provincial University Key Lab of Child Cognitive Development and Mental Health, Yancheng Teachers University, Yancheng 224002, China

**Keywords:** social adaptation, parenting styles, network analysis, rural left-behind children

## Abstract

Although prior research has typically examined the relationship between parenting styles and left-behind children’s social adaptation using broad categories without identifying directly linked dimensions, the present study employed network analysis to conceptualise both as interconnected dimensional networks. Methods: A total of 2452 children (713 left-behind, 1739 non-left-behind) were included. Results: (1) The results revealed that interpersonal adaptation and learning adaptation were core dimensions in the SA networks of both groups. (2) In the combined PS-SA network for left-behind children, the core dimensions were interpersonal adaptation, learning adaptation, father’s rejection, and mother’s rejection; for non-left-behind children, they were interpersonal adaptation, learning adaptation, mother’s rejection, and mother’s emotional warmth. (3) Network comparisons further indicated that the connections between father’s rejection and interpersonal adaptation, father’s rejection and learning adaptation, and father’s emotional warmth and learning adaptation were stronger in the left-behind group, whereas the connections between mother’s emotional warmth and positive emotional adaptation, and between interpersonal adaptation and learning adaptation, were stronger in the non-left-behind group. Conclusions: these findings visualise and specify how distinct parenting dimensions relate to different facets of social adaptation, offering parents and schools potential targets for adaptation education tailored to left-behind children.

## 1. Introduction

Left-behind children (LBC) are individuals under the age of 17 whose parents work in distant locations, resulting in their care being provided by a single parent or in the absence of direct guardianship. As of 2020, the number of such children in China reached 41.77 million (National Bureau of Statistics, 2020). Research indicates that these children often experience neglect and discrimination (Lan et al., 2018; J. Zhao et al., 2021), report a diminished sense of security, and are more susceptible to emotional disorders such as depression, anxiety, and loneliness (Ling et al., 2012; Fellmeth et al., 2018; M. L. Li et al., 2019). Furthermore, studies have shown that they may exhibit maladaptive social behaviors, including social withdrawal and aggression (Hu et al., 2018; Dong et al., 2022). These challenges in social adaptation have consequently garnered significant public and scholarly attention.

Social adaptation refers to the ongoing adjustment of an individual’s physical and mental state within dynamic social contexts, with the aim of achieving alignment with the current environment (Peng & Dai, 2023). According to the model of social competence, individuals acquire age-appropriate behaviors while living through continuously changing fields (Greenspan & Granfield, 1992); for school-age children, this adaptation should encompass several dimensions, including academic adaptation, life adaptation, emotional adaptation, interpersonal adaptation, and cognitive adaptation.

Presently, research on the social adaptation of left-behind children primarily exists in two orientations. The first orientation categorizes left-behind children into heterogeneous groups based on their response patterns in specific dimensions of social adaptation, further exploring the differential patterns among these categories (J. Zhao et al., 2019; Han et al., 2026). For instance, Li et al. employed latent class analysis to classify left-behind children into three types: well-adapted, those facing adaptation difficulties, and those exhibiting behavioral impulsivity, based on emotional and behavioral issues (Z.-H. Li et al., 2014). The study identified significant differences among the categories concerning gender and grade. The second research orientation evaluates the adaptation levels of left-behind children through a comprehensive score of social adaptation, enabling a comparison of adaptation differences among various groups (Fan et al., 2009; Hou et al., 2014). For instance, Fellmeth et al. (2018) conducted a meta-analysis comparing the overall social adaptation levels of left-behind and non-left-behind children (NLBC) across 111 studies, revealing that left-behind children generally exhibit more anxiety, depression, and other maladjustment problems.

However, the former orientation focuses only on a single dimension of adaptation and fails to adequately account for the interactions among different dimensions. The latter relies on traditional latent variable models, which assume that the latent variable serves as the common cause of observed variables within the same dimension, and that all correlations among observed variables are fully explained by the latent variable. That is, it assumes that all dimensions are equally influenced by the same latent variable, thereby overlooking the possibility that different dimensions may vary in importance. Consequently, neither research orientation can effectively identify the key dimensions of social adaptation in the developmental process of left-behind children.

In light of the limitations observed in prior studies, researchers have advocated for examining various social adaptation challenges—such as depression—through the lens of complex systems (Borsboom, 2008; Borsboom & Cramer, 2013). Using graphical model visualizations, they conceptualized social adaptation as a multidimensional complex network, where nodes and edges represent constituent elements and their interrelations (Borsboom & Cramer, 2013). This methodology is known as network analysis.

Specifically, this approach conceptualizes various dimensions of social adaptation as network nodes, with the lines between nodes representing the relationships among these dimensions. The thickness of each line indicates the strength of the correlation between nodes, while node centrality metrics reflect the importance of each dimension within the network (Opsahl et al., 2010). Network analysis emphasizes the interconnections among the different dimensions of social adaptation, using statistical modeling to highlight the most significant ones. This method effectively compensates for the limitations of traditional latent variable models, which often struggle to capture the relational patterns among social adaptation dimensions (Cai et al., 2020). Consequently, it provides a novel perspective for identifying key adaptive dimensions in children’s development (Borsboom, 2017). Therefore, the first objective of this study is to construct a network structure of social adaptation and to compare the differences in this network structure between left-behind children and non-left-behind children.

Moreover, according to ecological systems theory, the family constitutes the most critical component within the micro-environmental system (Bronfenbrenner, 2013). Within the family context, parenting style serves as a central factor significantly influencing the social adaptation of left-behind children (Fellmeth et al., 2018). Research indicates that positive parenting practices—such as emotional warmth—are associated with reduced anxiety, depression, loneliness, and behavioral problems, while also promoting greater happiness among these children (Carlo et al., 2011; Khaleque, 2013; Butterfield et al., 2021; Q. Zhang, 2025). In contrast, negative parenting styles marked by rejection or over protection have been correlated with poorer social adaptation outcomes (Cong et al., 2020; Schiff et al., 2021; Wu & Su, 2024). Furthermore, studies suggest that parenting styles not only directly affect the social adaptation of left-behind children but also indirectly predict it through mediating variables such as self-control and parent–child conflict (Pan et al., 2021; R. P. Zhang et al., 2023).

Although a substantial body of theoretical and empirical research has demonstrated that parenting styles exert significant and profound influences on the social adaptation of left-behind children. However, existing studies still exhibit two major limitations. First, the majority of relevant research has focused exclusively on maternal parenting behaviors (Song et al., 2024; Tancred & Greeff, 2015). For instance, Hoeve et al. conducted a meta-analysis of 161 studies and discovered that less than 20% of them specifically examined fathers’ parenting behaviors (Hoeve et al., 2009). Family systems theory posits that maternal and paternal parenting styles are interdependent and mutually influential rather than independent (Cox & Paley, 1997; Masten, 2018). Therefore, it is essential to simultaneously consider the effects of both fathers’ and mothers’ parenting styles on the social adaptation of rural left-behind children within in a unified model.

Secondly, while prior studies have predominantly relied on regression analysis to examine the influence of parenting styles on social adaptation, they often overlook the specific mechanisms through which parenting practices directly predict adaptive outcomes (López-Soler et al., 2009; Schoeps et al., 2020). The use of network analysis can more intuitively uncover how parenting styles are associated with social adaptation, thereby facilitating the design of targeted intervention strategies (Borsboom, 2017). Therefore, the second objective of this study is to construct a network structure of parenting styles and social adaptation (PS-SA), and to compare the differences in this PS-SA network between left-behind and non-left-behind children.

## 2. Materials and Methods

### 2.1. Participants

A total of 2919 children participated in the study. After excluding participants due to incomplete data or identical selecting responses (i.e., selecting the same number), the final sample comprised 2452 participants, yielding a valid response rate of 84.00%. The sample was approximately evenly distributed by gender, consisting of 1095 boys (48.9%) and 1143 girls (51.1%). Participants were aged between 9 and 16 years and were categorized into two groups: left-behind children (*n* = 713; age range: 9–16; Mage = 12.36, SD = 2.93) and non-left-behind children (*n* = 1739; age range: 9–16; Mage = 12.03, SD = 3.08). The gender distribution was similar in both groups, with 48.7% females among left-behind children and 51.5% among non-left-behind children.

### 2.2. Procedure

For the recruitment of both left-behind and non-left-behind children, we selected nine primary and secondary schools situated in the rural regions of Yancheng, Lianyungang, and Taizhou in Jiangsu Province, China. Data collection took place in December 2022, during which participants completed the questionnaire in approximately 20 to 30 min. In accordance with the guidelines established by the ethics committee, participants provided informed consent. We obtained research consent from school leaders and head teachers, while ensuring parental informed consent for participants aged 15 years or younger. This study received approval from the ethics committee of Yancheng Teachers University. All procedures adhered to the ethical standards set forth by the responsible committee on human experimentation and complied with the Helsinki Declaration.

### 2.3. Measurements

#### 2.3.1. Social Adaptation of Rural Children

The social adaptation of rural children was assessed with the Rural Children Social Adaptation Questionnaire (RCSAQ; Dai, 2019). The scale includes five dimensions: interpersonal adaptation (e.g., “Many classmates in my class are very kind to me”), learning adaptation (e.g., “I sincerely love learning”), positive emotional adaptation (e.g., “I can manage my negative emotions well”), cognitive adaptation (e.g., “I think it is normal for classmates to often argue about certain issues”) and life adaptation (e.g., “At home, I regularly do chores”), comprising a total of 26 items on a 5-point Likert scale ranging from 1 (not like me at all) to 5 (completely like me). Each dimension represents a positive aspect, and higher scores indicate a higher level of social adaptation for the rural children. This scale has demonstrated good reliability and validity in previous studies with Chinese children (Peng & Dai, 2023). The Cronbach’s α coefficient for the scale was 0.89.

#### 2.3.2. Parenting Styles

Parenting styles was assessed with Chinese version of the short-form Egna Minnen av Barndoms Uppfostran (s-EMBU; Jiang et al., 2010). The s-EMBU consisted of two subscales: one for fathers and one for mothers. Each subscale is further divided into three dimensions: emotional warmth (e.g., “My parents always try to encourage me to be the best”), rejection(e.g., “My parents often treat me in an embarrassing way”), and over-protection (e.g., “My parents forbid me from doing things that other kids can do because they’re worried I might get hurt”). Emotional warmth is considered a positive dimension, while rejection and over-protection are viewed as negative dimensions. In total, the overall scale comprises a total of 42 items, with each subscale contains 21 items that are identical in content. Each item was rated on a 4-point Likert scale, ranging from 1 = strongly disagree to 4 = strongly agree. A higher score indicates a higher tendency for the child’s father or mother to exhibit this particular parenting style. This scale has demonstrated good reliability and validity in previous studies with Chinese children (Song et al., 2024). The Cronbach’s α coefficient for the scale was 0.91.

### 2.4. Statistical Analyses

In this on-site questionnaire study, participants with over 10% missing item-level data were excluded; all remaining items had missing rates below 0.5%, and given the homogeneous sample and low missing proportion, mean imputation was considered appropriate (Shan et al., 2023). In this study, SPSS version 25.0 was used for descriptive statistical analysis, internal consistency reliability test and correlation analysis, and R 4.3.1 in RStudio 1.2.5033 was used to estimate PS-SA network structure and network comparison. The network analysis approach follows the standard guidelines published by Epskamp (Epskamp et al., 2018).

#### 2.4.1. Network Estimation

The network structure of continuous variables was estimated using the EBICglasso function from the qgraph package (Epskamp et al., 2012). In the network diagram, each node in the network represents an individual dimension of the questionnaire that captures a particular symptom. The relationships among dimensions are illustrated as edges. Positive correlations are shown with green edges, whereas negative correlations are indicated by red edges. A Gaussian Graphical Model (GGM) was utilized to assess these associations, which is appropriate for continuous data (Friedman et al., 2008). The Extended Bayesian Information Criterion Graphical Lasso (EBICglasso) algorithm was implemented, using a tuning parameter set to 0.5 (Epskamp et al., 2018). This methodology improves the detection of genuine network links while minimizing the influence of weaker associations. The evaluation of each node’s predictability was conducted with the “mgm” package, which measures the extent to which a node’s variance can be accounted for by its links within the network (Haslbeck & Waldorp, 2020). Additionally, the “ggplot2” package was employed to enhance the network’s visualization (Ognyanova, 2019). Furthermore, to enhance visualization across networks, we utilized the averageLayout function from the qgraph package.

#### 2.4.2. Centrality Estimation

To assess node importance in the network, we computed Expected Influence (EI) and Bridge Expected Influence (BEI) using the R 4.3.1 packages “qgraph” and “networktools”, identifying central and bridge nodes (Epskamp et al., 2018; Jones et al., 2021). EI was chosen for its better performance in networks with both positive and negative edges compared to other centrality indices (McNally, 2021). High EI reflects stronger positive associations with other nodes and greater overall network influence (Jones et al., 2021; Robinaugh et al., 2016). High-BEI nodes serve as crucial connectors between symptom clusters and may facilitate the development or persistence of other dimensions or disorders (Jones et al., 2021).

#### 2.4.3. Network Accuracy and Stability

The evaluation of network stability and accuracy was conducted utilizing the “bootnet” package (Epskamp et al., 2018). We executed 1000 bootstrap samples to estimate the confidence intervals (CIs) at 95% for edge weights and examined the differences among edges and nodes. A narrower CI at 95% reflects greater precision in the edge weights within the network. The stability of the node centrality indices was evaluated through the case-dropping bootstrap method, which indicates the highest permissible percentage of data that may be omitted without jeopardizing stability. The Correlation Stability Coefficient (CS) was computed, where a CS score of ≥0.25 is considered satisfactory, while values exceeding 0.5 are regarded as preferable (Epskamp et al., 2018).

#### 2.4.4. Network Comparison

The “NetworkComparisonTest” was used to compare the networks of rural left-behind children and non-rural left-behind children. Three tests were conducted to evaluate these differences: a test for network structure invariance, a test for global strength invariance, and a test for edge strength invariance (Van Borkulo et al., 2023). The network structure invariance test assessed variations in the strength of the maximum edge within the network; the global strength invariance test assessed differences in the total edge strength; and the edge strength invariance test examined variances in specific edges within the network.

## 3. Results

### 3.1. Descriptive Statistics and Correlation Analyses

First, SPSS 26.0 was employed to assess the normality of the total scores for each variable. The results indicated that the data approximated a normal distribution, thereby satisfying the assumptions for parametric statistical analyses. Subsequently, independent-samples *t*-tests were conducted to examine differences in parenting styles and social adaptation between left-behind children and non-left-behind children.

As indicated in Table 1, Significant differences in parenting styles were found between left-behind children and non-left-behind children on the MR, MEW, and FEW dimensions. left-behind children scored higher on MR but lower on MEW and FEW compared to on-left-behind children. No significant differences were observed on MO, FR, or FO dimensions. In social adaptation, left-behind children scored significantly lower on IA, LA, PEA, and CA dimensions, while no difference was found on AL.

**Figure 1:**
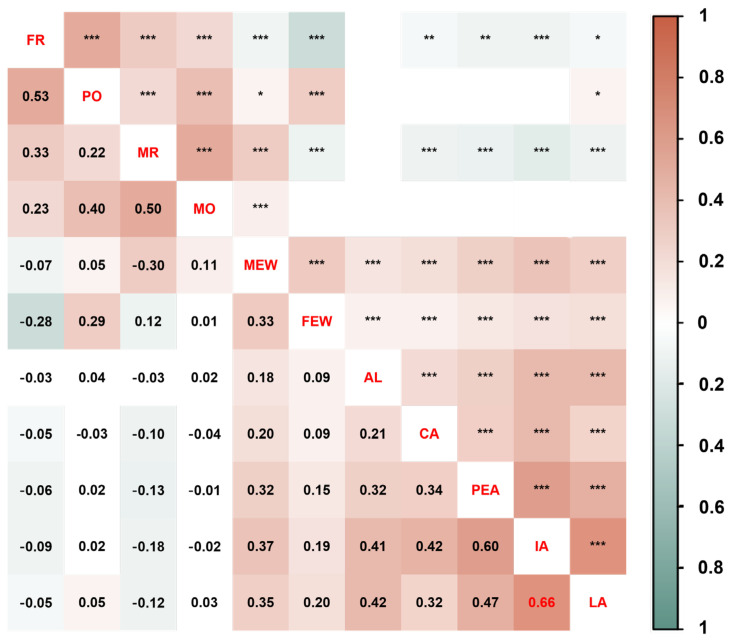
Heat map correlations. (1) The lower triangular matrix displays the correlation coefficients, with each value indicating Spearman’s c correlation coefficient. Only those coefficients that are statistically significant (*p* < 0.01) are included in the lower triangular matrix. (2) The upper triangular matrix illustrates the significance levels (* *p* < 0.05, ** *p* < 0.01, *** *p* < 0.001), while the empty boxes indicate coefficients do not survive this correction.

The correlation results are presented in Figure 1, FR, MR were significantly negatively associated with IA, LA, PEA and CA. MEW and FEW were significantly positively associated with IA, LA, CA, PEA, adaptation to life (AL). MOP and FOP did not show significant relationships with IA, LA, CA, and AL. Additionally, the correlation between MR, FR, and AL was also found to be non-significant.

### 3.2. Social Adaptation Networks

#### 3.2.1. Network Estimation

**Figure 2:**
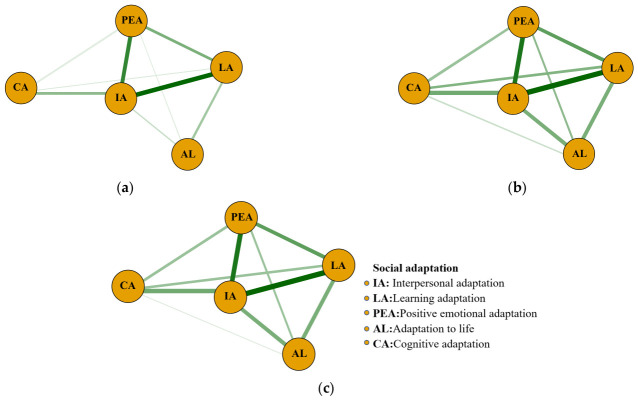
Network structure of social adaptation. Notes: (1) (**a**–**c**) respectively represent the social adaptation network structure of Overall sample of children, left-behind children, and non-left-behind children. (2) Each node represents a dimension of social adaptation. (3) Green lines mean positive connections, and the edge thickness indicates correlation strength. (4) To facilitate visual comparisons of the network structures across different groups, this study employs the average layout function, ensuring that the positions of identical nodes remain consistent.

To comprehend the network structure of social adaptation (SA), we estimated three regularized networks based on left-behind children (Figure 2a), non-left-behind children (Figure 2b), and overall children (Figure 2c), encompassing all 5 dimensions. This resulted in a total of 10 edges (5 × (5 − 1)/2). In the social adaptation network of the overall sample of children and the non-left-behind children sample, there are 10 edges with non-zero weights (mean weight 0.17 and 0.22). The social adaptation network for left-behind children includes 9 edges with non-zero weights (mean weight 0.16). Figure 2 demonstrates the significant strength of internal connections within each dimension. It is noteworthy that in all three SA networks, IA and LA consistently exhibit the strongest connectivity.

#### 3.2.2. Centrality Estimation

**Figure 3:**
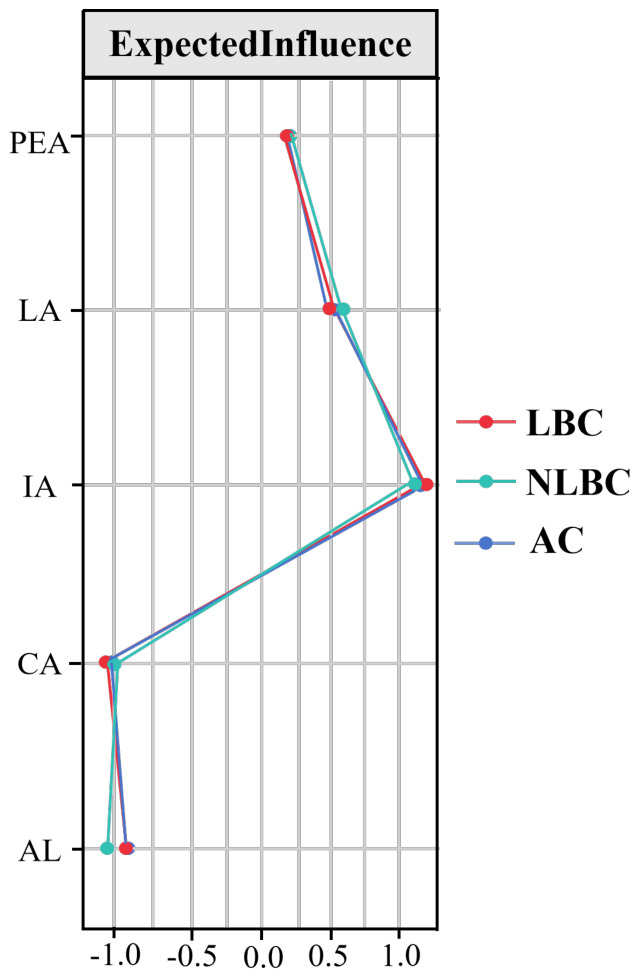
Centrality plots for three SA networks. The *X*-axis represents the centrality indices as standardized z-scores, indicating that a higher estimate corresponds to greater centrality of the item, while the *Y*-axis displays the 11 variables.

Figure 3 illustrates the expected influence centrality of each dimension across the three SA networks. The results indicate that both IA (EI = 1.53\1.50\1.49) and LA (EI = 0.50\0.52\0.53) demonstrate the highest expected influence in all three networks, showing significant differences in strength centrality compared to other dimensions.

#### 3.2.3. Network Accuracy and Stability

The results of edge-weight bootstrapping indicate that the three network estimations were moderately accurate. The CS coefficients for expected influence and bridge expected influence in all two networks are above 0.50, suggesting that the overall network stability was excellent (see Supplementary Material Figure S1).

#### 3.2.4. Network Comparison

Network comparisons between left-behind children and non-left-behind children were conducted through three tests. First, the network structure invariance test, revealed no significant differences in the overall network structure between the two groups (*p* = 0.08 > 0.05), indicating similar structures. Second, the global strength invariance test, also showed no significant differences in the strength of SA networks (*p* = 0.15 > 0.05). Third, the edge invariance test demonstrated significant differences in two edges between left-behind children’ and non-left-behind children’ networks. Notably, the edges linking IA and AL exhibited greater expected influence in the left-behind children’ networks compared to non-left-behind children’ networks. In contrast, the edges linking IA and PEA exhibited greater expected influence in the non-left-behind children’ networks compared to left-behind children’ networks. For a comprehensive overview of all significant differences identified in the edge invariance test, please refer to the Supplementary Information (Table S1).

### 3.3. Combined Networks

#### 3.3.1. Network Estimation

**Figure 4:**
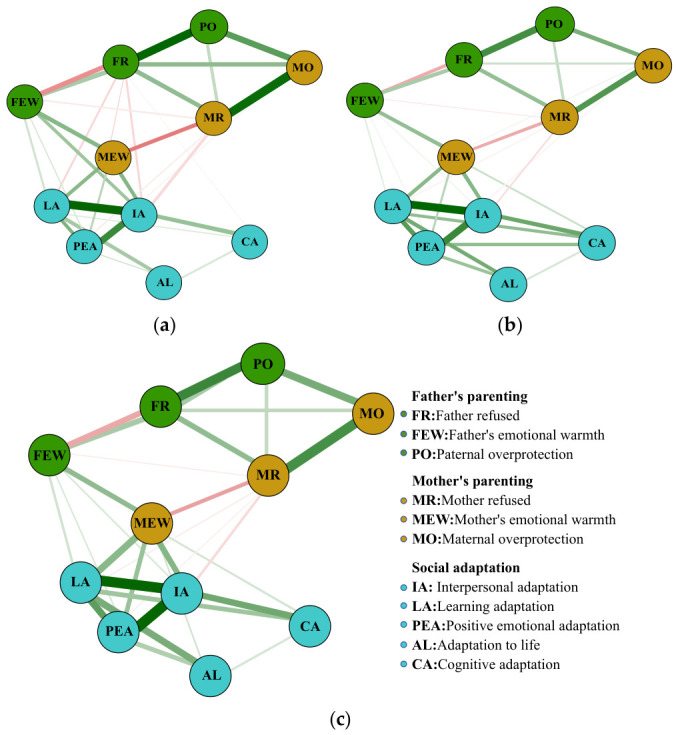
Network structure of parenting styles–social adaptation. Notes: (1) (**a**–**c**) respectively represent the social adaptation network structure of children as a whole, left-behind children, and non-left-behind children. (2) Each node represents a dimension of parenting styles and social adaptation. (3) Green lines mean positive connections, red lines mean negative connections, and the edge thickness indicates correlation strength. (4) To facilitate visual comparisons of the network structures across different groups, this study employs the average layout function, ensuring that the positions of identical nodes remain consistent.

Given the marked differences in parenting styles and social adaptation between left-behind and non-left-behind children, this study further examined variations in their network structures. We constructed three regularized partial correlation networks—for left-behind children (Figure 4a), non-left-behind children (Figure 4b), and the overall sample (Figure 4c)—incorporating all 11 dimensions, resulting in 55 possible edges. The overall and non-left-behind networks each contained 29 non-zero edges with a mean weight of 0.05, while the left-behind children’s network had 32 non-zero edges and a higher mean weight of 0.06. Notably, the strongest undirected edge connecting the parenting styles and social adaptation sub-network in the left-behind children network was between IA and MEW (edge weight = 0.12). In contrast, the non-left-behind children network exhibited the strongest edge between LA and MEW (edge weight = 0.13), followed by the edge between PEA and MEW (edge weight = 0.11).

#### 3.3.2. Centrality Estimation

**Figure 5:**
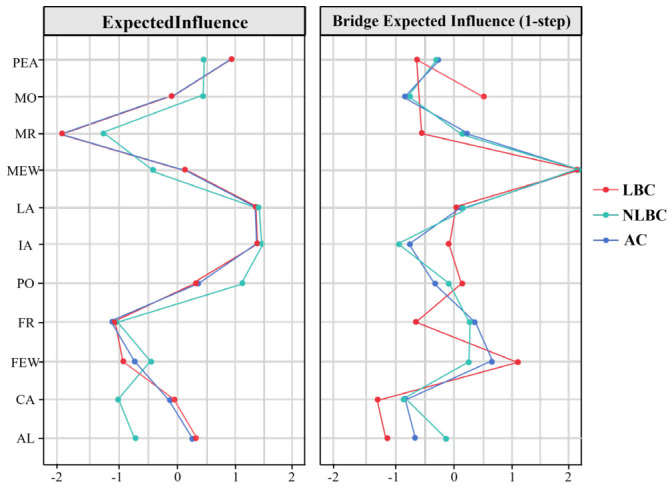
Centrality plots for three PS-SA networks. The *X*-axis represents the centrality indices as standardized z-scores, indicating that a higher estimate corresponds to greater centrality of the item, while the *Y*-axis displays the 11 variables.

Prior to constructing the network structure, each dimension was standardized due to the differing scoring methods of the parenting style and social adaptation questionnaires. As shown in Figure 5, based on expected influence values, the top 20% of nodes across all three network structures were IA (EI = 1.19 for all children, 1.16 for left-behind, 1.20 for non-left-behind) and LA (0.94, 0.92, 0.92, respectively). Regarding bridge expected influence, both MEW and FEW ranked among the top 20% of nodes in all three PS-SA networks, indicating they serve as critical bridges connecting the entire network. Furthermore, the strongest correlation in all three SA networks was between interpersonal adaptation (IA) and adaptation to life (AL), followed by the connection between IA and positive emotional adaptation (PEA).

#### 3.3.3. Network Accuracy and Stability

The results of edge-weight bootstrapping indicate that the two network estimations were moderately accurate. The CS coefficients for strength, closeness, and betweenness, and expected influence in all three networks are above 0.50, suggesting that the overall network stability was excellent (see Supplementary Material Figure S2).

#### 3.3.4. Network Comparison

Network comparisons between left-behind children and non-left-behind children were conducted through three tests. First, the network structure invariance test revealed no significant differences in the overall network structure between the two groups (M = 0.14, *p* = 0.21), indicating similar structures. Second, Secondly, the test for global strength invariance revealed no significant difference in the overall network strength between the two groups (global strength for LBC = 4.96; NLBC = 6.34; S = 1.38, *p* = 0.16). This result confirms that the network density in terms of overall strength is comparable between left-behind children and non-left-behind children. Third, the edge invariance test demonstrated significant differences in nine edges between left-behind children’ and non-left-behind children’ networks. Notably, the connections between FR and IA, FR and LA, as well as FEW and LA were significantly stronger in left-behind children compared to their non-left-behind counterparts (*p* < 0.05). On the other hand, the connections between MEW and PEA, IA and LA, were significantly stronger in non-left-behind children compared to their left-behind counterparts (*p* < 0.05). For a comprehensive overview of all significant differences identified in the edge invariance test, please refer to the Supplementary Information (Table S1).

## 4. Discussion

This study is the first to utilize network analysis in examining the core dimensions of social adaptation among left-behind children and non-left-behind children, as well as the relationship between parenting styles and social adaptation. Firstly, This study identified the central dimensions of SA networks across different groups. Secondly, this study identified the central dimensions of PS-SA networks across different groups. Lastly, this study conducted three tests to compare the differences in SA networks and PS-SA networks between left-behind children and non-left-behind children. By employing a network approach, the study provides new insights into which dimensions have the most significant impact on children’s social adaptation and how parenting style and social adjustment dimensions are interconnected within the network. These findings can inform the development of precise and effective interventions.

### 4.1. Descriptive Statistical Analysis

To begin with, descriptive statistics indicated differences in social adjustment and parenting styles between left-behind and non-left-behind children. Specifically, left-behind children scored significantly lower on maternal emotional warmth (MEW) and paternal emotional warmth (FEW) compared to non-left-behind children, while scoring significantly higher on maternal rejection (MR). This indicates that left-behind children perceive less positive parenting behaviors and more negative rejecting behaviors from their parents. Previous studies indicate that when a parent migrates for work, reduced direct interaction leads to inadequate care for left-behind children (X. Zhao et al., 2023). Weaker parent–child communication also hinders timely awareness of children’s needs, potentially intensifying perceived parental rejection (Adumitroaie & Dafinoiu, 2013; Z. Liu et al., 2024). Notably, left-behind children in this study scored significantly higher on maternal rejection, but showed no significant difference in paternal rejection. This pattern may be attributed to the high proportion of families in the sample where the father had migrated for work. In these households, mothers often become solely responsible for child care and education after the father’s departure. The increased burden tends to generate greater pressure and parent–child conflict, leading to elevated levels of rejecting behavior—a finding consistent with existing literature (Z. Liu & Chen, 2018).

The study also revealed significant associations between parenting styles and social adaptation. Specifically, positive parenting styles exhibited a significant positive correlation with social adaptation, whereas negative parenting styles demonstrated a significant negative correlation with social adaptation—findings that align with existing research (Fellmeth et al., 2018; Napolitano et al., 2011). Positive parenting fosters a nurturing and supportive environment for children, which enhances their internal psychological resources, life skills, and social abilities, thereby promoting their social adaptation (Carroll, 2022). In contrast, rejecting or overprotective parenting can undermine children’s self-confidence, independence, resilience, and coping abilities, potentially impairing their self-regulatory behaviors and increasing the risk of problematic behaviors (Kang et al., 2024).

### 4.2. Social Adaptation Networks Analysis

Network analysis results reveal that interpersonal adaptation (IA) and learning adaptation (LA) were the core dimensions in three SA network structures, highlighting the importance of interpersonal adaptation and learning adaptation for both groups of children. These dimensions may play an important role in social adaptation and can have implications for various other areas. Previous studies have found that children who struggle with interpersonal or academic adjustment may experience challenges in emotional well-being, life adjustments, and may exhibit maladaptive behaviors (Moilanen et al., 2010). Specifically, those with poor interpersonal adjustment may exhibit feelings of loneliness, social withdrawal, and aggression (Jin et al., 2023; Chen et al., 2015), while those with academic struggles may face internalizing issues like self-doubt, anxiety, and depression (Deighton et al., 2018). These difficulties can hinder their ability to effectively adapt to their current social environment.

Furthermore, this study found that the connection between interpersonal adaptation (IA) and adaptation to life (AL) showed the strongest correlation in all three SA networks, followed by the connection between interpersonal adaptation (IA) and positive emotional adaptation (PEA). These results indicate a significant bidirectional relationship between interpersonal adaptation, learning adaptation, and positive emotional adaptation, which supports the idea of predictive relationships in the developmental cascade theory (Masten et al., 2005). Consistent results have also been found in college students by H. Liu and Li (2024).

We also examined the network variations in the SA network between rural left-behind children and non-rural left-behind children. The findings revealed no significant differences in network structure invariance and global strength invariance between the two groups, but a notable distinction in edge invariance. Specifically, the connections between nodes in the SA network of rural left-behind children were weak, indicating a lack of robust interactions across various dimensions of social adaptation within this group. In line with the developmental cascade theory of social adaptation, positive development in one dimension should positively influence other dimensions of adaptation (Masten & Cicchetti, 2010). Based on the findings of this study, this highlights that the limited interconnection among different social adaptation dimensions in left-behind children impedes the establishment of a positive development cycle.

### 4.3. Combined Networks

Network analysis revealed that the core dimensions in the PS-SA network for left-behind children were interpersonal adaptation (IA), learning adaptation (LA), and paternal overprotection (PO), whereas for non-left-behind children, the core dimensions were interpersonal adaptation (IA), learning adaptation (LA), and Positive emotional adaptation (PEA). This difference highlights two key phenomena: First, left-behind children experience greater paternal overprotection, which may arise from the fact that in left-behind families, fathers often work away from home while mothers assume daily caregiving responsibilities. This physical absence may cause fathers to develop a compensatory mindset, making them more inclined to indulge their children’s demands. Second, non-left-behind children exhibit better development in positive emotional adaptation, likely due to the emotional guidance and support they receive from both parents, enabling them to develop emotional regulation and social adaptation skills more effectively (Fan et al., 2009).

According to the analysis of bridge expected influence, paternal emotional warmth exhibited the highest bridge expected influence indicator, followed by maternal emotional warmth. Among both left-behind and non-left-behind children, paternal emotional warmth showed the strongest association with learning adaptation, while maternal emotional warmth was more prominently linked to interpersonal adaptation. This suggests that positive paternal parenting styles contribute to enhancing children’s adaptive abilities in the academic domain. Existing research similarly indicates that a paternal parenting style characterized by high warmth and high control tends to predict better academic achievement in children (Pong et al., 2005). On the other hand, mothers, by sensitively and promptly responding to children’s needs, can provide more adequate emotional support, thereby positively influencing the development of children’s cooperative behavior and social competence (Mahoney & Perales, 2005).

Differences were observed in the networks of left-behind children compared to their non-counterparts. Specifically, we found that rural left-behind children show significantly stronger associations between Father refused (FR) and interpersonal adaptation (IA), Father refused (FR) and learning adaptation (LA), as well as father’s emotional warmth (FEW) and learning adaptation (LA). Conversely, non-left-behind children exhibit significantly stronger associations between Mother’s emotional warmth (MEW) and Positive emotional adaptation (PEA), as well as between interpersonal adaptation (IA) and learning adaptation (LA). These findings indicate that left-behind children might struggle with learning and interpersonal adjustment issues when experienced higher levels of paternal rejection. Previous studies have also shown that paternal rejection can lead to a lack of social skills in children, resulting in difficulties in interpersonal and academic adjustment (Rohner & Britner, 2002). Conversely, the presence of emotional warmth from fathers has been linked to better academic adaptability in children, possibly due to variations in fathers’ involvement in their children’s lives and education (Ki, 2020). Left-behind children often have limited communication with their fathers and are more likely to experience conflicts over academic matters, which can impede their academic adjustment. Moreover, mothers of non-left-behind children appear to exert a more significant influence on their children’s emotional well-being. This may be attributed to the fact that these mothers have more time and energy to dedicate to addressing their children’s emotional needs, thereby helping them to effectively manage negative emotions and moods.

Utilizing network analysis, this study reveals the social adaptation structures of both left-behind and non-left-behind children and examines the association patterns between parenting styles and dimensions of social adaptation. It offers new insights into the challenges and mechanisms involved. The findings can assist parents and schools in developing more targeted adaptive education, particularly by emphasizing interpersonal and learning adaptations—important areas that require nurturing through both family and school environments. Importantly, the study notes that left-behind children often perceive paternal overprotection and exhibit limited cross-domain adaptation transfer. This underscores the necessity for fathers to engage more actively, enhancing communication and guidance in worldviews and interpersonal skills to effectively improve these children’s social adaptation.

Despite the novelty of our study and the valuable findings presented, these limitations should be acknowledged. The primary limitation of this study is that the development of network models relies on cross-sectional studies and group-level data, which may not adequately elucidate the causal associations between nodes. Therefore, longitudinal intra-individual analyses, such as dynamic networks (Bos et al., 2017), are necessary to complement our findings. The second limitation of this study is the reliance on self-report measures, which may be subject to self-report biases (Caputo, 2017). Future research could consider incorporating clinician-administered interviews to assess problematic or addictive behaviors. The third limitation of this study is its exclusive focus on the relationship between parenting styles and social adaptation. However, children’s social adaptation is a multifaceted concept that is developmentally specific. It is influenced not only by family factors but also by various elements within the school and broader social environments (Fan et al., 2023). Future research should consider simultaneously investigating the interaction mechanisms of multiple factors on children’s social adaptation.

## 5. Conclusions

To the best of our knowledge, this is the first study to utilize network analysis technology in comparing the social adaptation network structural characteristics of left-behind children and non-left-behind children, as well as to examine the influence of parenting styles on social adaptation. This study enhances the understanding of the interrelationship between parental rearing styles and the social adjustment. First, within the SA network, we found that the core dimensions of left-behind children and their non-left-behind counterparts are identical, with both groups exhibiting IA and LA. This suggests that these two dimensions serve as the most effective indicators of children’s social adaptation. A comparison of the networks of both groups revealed similar network structures and global strengths, yet notable differences in specific dimension associations. Second, within the PS-SA network, we found that the core dimensions of left-behind children differ from those of non-left-behind children. Specifically, interpersonal adaptation (IA), learning adaptation (LA), and paternal over protection (PO) comprise the core dimensions of the left-behind children network, whereas, interpersonal adaptation (IA), learning adaptation (LA), and positive emotional adaptation (PEA) constitute the core dimensions of the non-left-behind children network. Although the networks of both groups displayed similar structures, they varied in global strength and specific dimension associations. The research findings tell us that social adaptation interventions for left-behind children should prioritize improving their interpersonal and learning adaptation, along with reducing paternal overprotection, to foster a more positive developmental trajectory.

## Figures and Tables

**Table 1:** Descriptive statistics for main variables.

Variable	Left-Behind (*n* = 713)	Non-Left-Behind (*n* = 1739)	All of Children (*n* = 2452)	t	*p*	Cohen’s d
	M ± SD	M ± SD	M ± SD			
MR	1.70 ± 0.62	1.74 ± 0.66	1.67 ± 0.60	2.48	0.001	0.62
MEW	2.97 ± 0.69	2.90 ± 0.72	3.01 ± 0.68	−3.50	<0.001	0.69
MO	2.34 ± 0.56	2.36 ± 0.57	2.34 ± 0.56	0.91	0.361	0.56
FR	1.67 ± 0.63	1.68 ± 0.63	1.65 ± 0.62	0.82	0.417	0.62
FEW	2.88 ± 0.72	2.85 ± 0.72	2.92 ± 0.72	−2.05	0.041	0.72
PO	2.31 ± 0.51	2.30 ± 0.54	2.31 ± 0.50	−0.30	0.766	0.51
IA	4.02 ± 0.69	3.98 ± 0.69	4.10 ± 0.68	−3.21	0.001	0.69
LA	3.94 ± 0.80	3.92 ± 0.82	4.02 ± 0.78	−2.76	0.001	0.80
PEA	3.89 ± 0.86	3.83 ± 0.89	3.98 ± 0.83	−3.99	<0.001	0.85
CA	4.23 ± 0.74	4.20 ± 0.76	4.29 ± 0.72	−2.51	0.012	0.73
AL	3.66 ± 0.93	3.68 ± 0.96	3.75 ± 0.92	−1.56	0.118	0.93

Notes: Interpersonal adaptation = IA, Learning adaptation = LA, Positive emotional adaptation = PEA, Cognitive adaptation = CA, Adaptation to life = AL, Mother’s emotional warmth = MEW, Father’s emotional warmth = FEW, Maternal overprotection = MO, Paternal overprotection = PO, Father refused = FR, Mother refused = MR.

## Data Availability

The raw data supporting the conclusions of this article will be made available by the authors on request.

## Supplementary Materials

The following supporting information can be downloaded at: https://www.mdpi.com/article/10.3390/bs16060857/s1, Figure S1: Results of SA networks accuracy and stability. A, B, and C respectively represent the CS coefficients of children as a whole, left-behind children, and non-left-behind children. Figure S2: Results of PS-SA networks accuracy and stability. A, B, and C respectively represent the CS coefficients of children as a whole, left-behind children, and non-left-behind children. Table S1: PS-SA network edge invariance test results.
