# Supplementary material for: The Relationship Between Social Adaptation and Parenting Styles in Left-Behind and Non-Left-Behind Children: A Network Analysis
Source: Behav Sci (Basel). 2026 May 27;16(6):857. doi: 10.3390/bs16060857 (PMC13295982; doi:10.3390/bs16060857)
Supplement: Supplementary file 1 [file behavsci-16-00857-s001.zip › behavsci-4312525-supplementary.pdf]

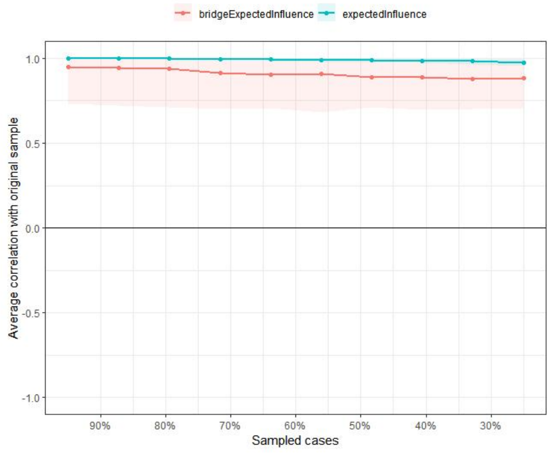

(A)

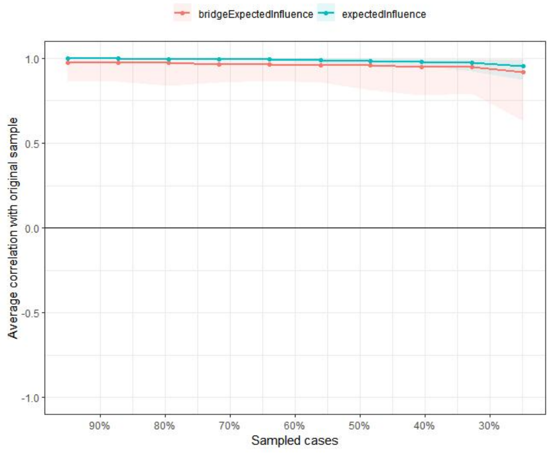

(B)

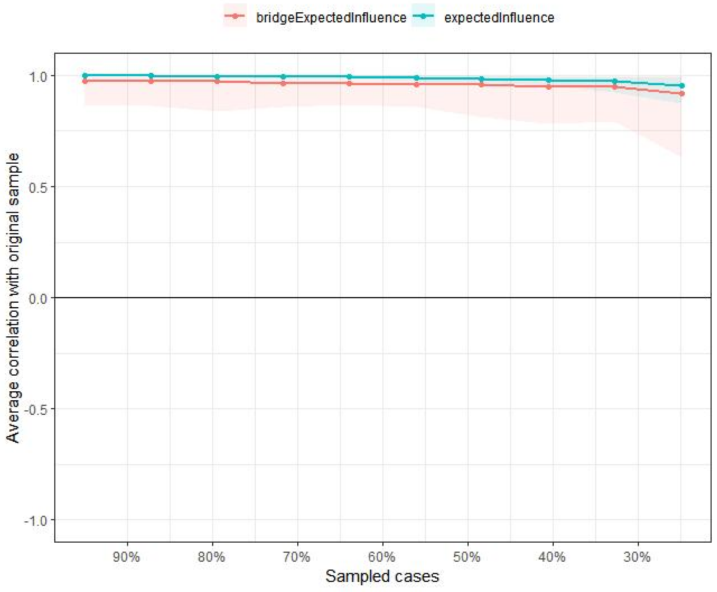

(C)

Figure S1 Results of SA networks accuracy and stability. A, B, and C respectively represent the CS coefficients of children as a whole, left-behind children, and non-left-behind children.

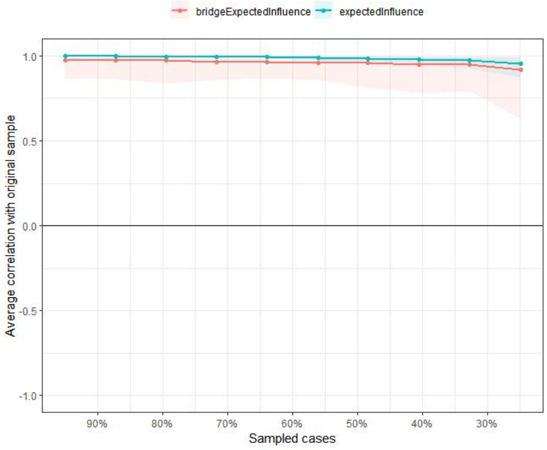

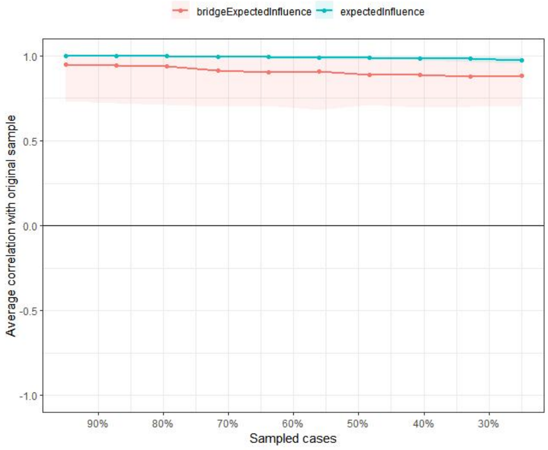

(A) **(B)**

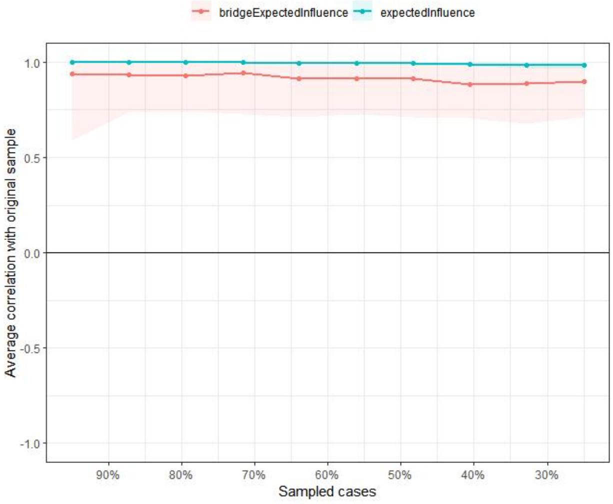

(C)

Figure S2 Results of PS-SA networks accuracy and stability. A, B, and C respectively represent the CS coefficients of children as a whole, left-behind children, and non-left-behind children.

Table S1. PS-SA network edge invariance test results

**Edge**	**Overall**	**LBC**	**NLBC**	**LBC vs NLBC (p value)**
IA-LA	0.447	0.438	0.440	0.970
IA-PEA	0.369	0.312	0.390	**0.049**
LA-PEA	0.077	0.117	0.060	0.317
IA-CA	0.205	0.197	0.206	0.871
LA-CA	0.040	0.000	0.057	0.168
PEA-CA	0.102	0.113	0.091	0.614
IA-AL	0.142	0.170	0.078	**0.039**
LA-AL	0.187	0.213	0.170	0.336

PEA-AL	0.073	0.071	0.066	0.921
CA-AL	0.025	0.031	0.011	0.490
IA-MR	-0.050	-0.033	-0.050	0.613
LA-MR	0.013	0.000	0.000	1.000
PEA-MR	0.000	0.000	0.000	1.000
CA-MR	0.000	0.000	0.000	1.000
AL-MR	0.034	0.000	0.041	0.316
IA-MEW	0.079	0.125	0.057	0.119
LA-MEW	0.113	0.075	0.127	0.228
PEA-MEW	0.066	0.059	0.110	0.208
CA-MEW	0.098	0.000	0.049	0.149
AL-MEW	0.006	0.000	0.020	0.247
MR-MEW	-0.355	-0.301	0.000	0.634
IA-MO	-0.005	0.000	-0.008	0.376
LA-MO	0.006	0.000	0.008	0.455
PEA-MO	-0.001	0.000	0.000	1.000
CA-MO	-0.017	0.000	-0.024	0.189
AL-MO	0.000	0.000	0.000	1.000
MR-MO	0.516	0.489	0.483	0.921
MEW-MO	0.297	0.200	0.277	0.168
IA-FR	-0.008	-0.012	-0.003	0.367
LA-FR	0.000	-0.011	0.014	**0.040**
PEA-FR	0.000	0.000	0.000	1.000
CA-FR	0.000	-0.019	0.000	0.059
AL-FR	0.000	-0.004	0.000	0.168
MR-FR	0.232	0.108	0.211	0.099
MEW-FR	0.137	0.000	0.116	0.158
MO-FR	-0.140	0.000	-0.122	0.129
IA-FEW	0.001	0.060	0.000	**0.010**
LA-FEW	0.044	0.087	0.022	**0.040**
PEA-FEW	0.002	0.000	0.004	0.722
CA-FEW	0.000	0.000	0.001	0.436
AL-FEW	0.000	0.000	0.000	1.000
MR-FEW	0.086	0.000	0.040	0.416
MEW-FEW	0.289	0.170	0.287	**0.030**
MO-FEW	-0.160	-0.025	-0.135	0.188
FR-FEW	-0.511	-0.387	0.224	**0.020**
IA-FO	0.000	0.000	0.000	1.000
LA-FO	0.001	0.000	0.018	0.188
PEA-FO	0.006	0.000	0.000	1.000

CA-FO	-0.019	-0.006	-0.009	0.950
AL-FO	0.004	0.000	0.004	0.505
MR-FO	-0.103	0.000	-0.005	0.347
MEW-FO	-0.137	0.000	-0.127	0.109
MO-FO	0.340	0.240	0.316	0.257
FR-FO	0.627	0.563	0.606	0.416
FEW-FO	0.530	0.388	0.528	**0.020**

Note: This table presents a comparative analysis of edges across various network structures. For example, it assesses whether there is a significant difference between the 'IA-LA' edge in the male population network and that in the female population network. This analysis facilitates the evaluation of differences in edges among different groups. The sections highlighted in bold indicate edges with significant differences.
